# Interaction of *starch branching enzyme 3* and *granule-bound starch synthase 1* alleles increases amylose content and alters physico-chemical properties in *japonica* rice (*Oryza sativa* L.)

**DOI:** 10.3389/fpls.2022.968795

**Published:** 2022-08-05

**Authors:** Kyu-Chan Shim, Cheryl Adeva, Ju-Won Kang, Ngoc Ha Luong, Hyun-Sook Lee, Jun-Hyeon Cho, HyunJung Kim, Thomas H. Tai, Sang-Nag Ahn

**Affiliations:** ^1^Department of Agronomy, College of Agriculture and Life Sciences, Chungnam National University, Daejeon, South Korea; ^2^Department of Southern Area Crop Science, Rural Development Administration, Miryang, South Korea; ^3^Crop Breeding Division, National Institute of Crop Science, Wanju-Gun, South Korea; ^4^LG Chem., Ltd., Seoul, South Korea; ^5^USDA-ARS Crops Pathology and Genetics Research Unit, Davis, CA, United States; ^6^Department of Plant Sciences, University of California, Davis, Davis, CA, United States

**Keywords:** amylose content, near-isogenic lines, physico-chemical trait, starch synthase gene, rice

## Abstract

Four near-isogenic lines (NILs) with different allele combinations of the *starch branching enzyme 3* (*SBE3*) and *granule-bound starch synthase 1* (*GBSS1*) were developed by crossing the *japonica* rice cultivars “Dodamssal” and “Hwayeong.” The associations between sequence variations in *SBE3* and *GBSS1*, and starch-related traits were investigated. These sequence variations led to changes in seed morphology, starch structure, starch crystallinity, amylopectin chain length distribution, digestibility, apparent amylose content (AAC), and resistant starch content (RS). *SBE3* and *GBSS1* showed genetic interaction in regulating AAC and RS. Gene expression profiling of panicle tissues revealed significant differences in expression levels of *GBSS1*, *SBE3*, and other starch-related genes among the four NILs, indicating that variations in *GBSS1* and *SBE3* changed the expression level of starch-related genes. These variations contributed to the changes observed in AAC, RS, and physico-chemical characteristics of the rice starch from the NILs.

## Introduction

Rice (*Oryza sativa* L.), one of the most important cereal crops in the world, is high in starch. Starch is the main energy-reserve polysaccharide in cereal crops, and is the chief source of carbohydrates in the human diet (Amagliani et al., 2016). Starch also has many technological, medical, and industrial uses (Miura et al., 2021). Rice starch is mainly composed of two biopolymers: amylose and amylopectin. Amylose is a linear molecule which is consisted of α (1–4) glucosidic chains, while amylopectin is highly branched glucan with α (1–6) glucosidic chains for a connection of linear chains (Yang et al., 2012).

Over the last few years, higher amylose content in staple crops has gained considerable interest due to its positive correlation with resistant starch (RS; Kim et al., 2018). RS is resistant to enzymatic hydrolysis (i.e., digestion) in the small intestine and is fermented by microorganism in the large intestine to produce short chain fatty acid (Englyst et al., 1982; Yang et al., 2016). RS-enriched cereals offer potential benefits for human health. RS consumption could lead to reduced glycemic and insulin responses and could lower the risk of developing type-II diabetes mellitus, obesity, and cardiovascular diseases (Zhou et al., 2016; Wang et al., 2017). High-amylose starches also exhibit different physico-chemical properties. Thus, understanding how these physico-chemical traits are controlled will be of practical value in many industrial food and non-food industries (Wang et al., 2017; Kim et al., 2018).

As the potential health benefits of RS-enriched cereals become more apparent, higher amylose rice mutants and varieties have been developed to address consumer demands. Several genes controlling amylose content have been cloned and characterized. The amylose in rice endosperm is mainly synthesized by granule-bound starch synthase I (GBSS1), which is encoded by the *Waxy* (*Wx*) gene on chromosome 6 (Sano, 1984). Three major *Wx* alleles have been identified: *wx*, *Wx^a^*, and *Wx^b^*. In the 5′ splicing site of intron 1, the *Wx^a^* allele harbors a G-SNP (Chr 6: 1,765,761) and is mainly found in *indica* rice whereas the *Wx^b^* allele harbors a T-SNP at this position and typically occurs in *japonica* rice (Cai et al., 1998; Yamanaka et al., 2004). The T/G SNP is associated with a variation of amylose content with the G allele (*Wx^a^*) corresponding to a higher expression level of *GBSS1* and increased amylose content (Sano et al., 1986). In addition, a non-synonymous SNP in Ex10-115 (C/T) is associated with gel consistency and a low rapid viscosity analysis (RVA) phenotype with most *indica* (*Wx^a^*) and *japonica* (*Wx^b^*) rices having a T (Serine) and a C (Proline), respectively (Chen et al., 2008; Zhang et al., 2019). Recently, the ancestral allele of *Waxy*, *Wx^lv^* (allele responsible for low viscosity and high amylose content), was isolated using a map-based cloning approach (Zhang et al., 2019). Although both *Wx^lv^* and *Wx^a^* possess a G SNP at 5′ splicing site of intron 1, *Wx^a^* harbors a T SNP at Ex10-115 whereas *Wx^lv^* carries a C SNP, which is mainly found in *japonica* rices (*Wx^b^*; Zhang et al., 2019). *Wx^lv^* allele expression level was higher than *Wx^b^* and was similar to *Wx^a^* due to the G-SNP at the 5′ splicing site in intron 1. Although the expression level of *Wx^a^* and *Wx^lv^* was similar, their eating and cooking qualities were significantly different due to the SNP at Ex10-115.

Amylopectin is a branched glucose polymer and is mainly synthesized *via* concerted reactions catalyzed by three types of biosynthetic enzymes: starch synthases (SSs), starch branch enzymes (SBEs), and debranching enzymes (DBEs; Wang et al., 2017). Among these enzymes, mutations in the SBE isozymes showed significant association with amylose content variation (Nishi et al., 2001; Satoh et al., 2003; Butardo et al., 2011; Yang et al., 2012; Kim et al., 2018; Adeva et al., 2020; Miura et al., 2021). In rice, three isoforms of SBE are present: BEI (SBE1 or BE1), BEIIa (SBE4 or BE2a), and BEIIb (SBE3 or BE2b). Among these isozymes, *SBE3* plays a major role in amylopectin synthesis, and a mutation in *SBE3* has been found to increase the amylose and RS content in the rice endosperm (Nishi et al., 2001; Yang et al., 2012; Adeva et al., 2020). Yang et al. (2012) identified an SNP (T/C) in the *SBE3* coding region resulting in a missense mutation (Leu-599-Pro) that was possibly responsible for high RS. This was confirmed by complementation (Yang et al., 2016). The same T/C SNP was also identified in *SBE3* of high amylose variety Dodamssal with high amylose and RS content (Adeva et al., 2020).

Recently, double mutant lines with mutations in or downregulation of amylose or amylopectin biosynthesis genes have been reported. The *beI*/*beIIb* double knockdown transgenic lines showed drastic increase in apparent amylose content (AAC) in an *indica* genetic background (Zhu et al., 2012). Asai et al. (2014) crossed two *japonica* rice mutants EM10 (*be2b*) and *e1* (*ss3a*) to generate a *ss3a*/*be2b* double null mutant line with 45% AAC. A higher amylose line (*be1be2b*) with an ultra-high level of RS was developed by crossing *BEI* and *BE2b* mutant lines (Miura et al., 2021). The *be1be2b* mutant had 51.7% AAC and 35.1% RS content. These reports showed that *SBE3* (*BE2b*) plays a crucial role in regulating amylose and amylopectin content. Understanding comprehensively the genetic interaction between starch synthase genes controlling amylose content will greatly contribute to breeding higher amylose rice varieties.

In this study, we developed four near-isogenic lines (NILs) with different allele combinations of *SBE3* and *GBSS1* derived from the cross between a Korean elite line Hwayeong and a high-amylose cultivar Dodamssal. The four NILs and two parental lines were evaluated for physico-chemical characteristics including seed morphology and starch structure, X-ray diffraction, starch viscosity, and amylopectin chain length distribution. Also, we analyzed the expression of the starch biosynthesis genes in the NILs to understand the interaction among genes with respect to control of amylose content. Our results indicate that *SBE3* and *GBSS1* alleles of Dodamssal synergistically contributed to an increase in the AAC and altered various physico-chemical characteristics. In addition, *SBE3* directly or indirectly regulates expression of the starch synthase and *ADP-glucose pyrophosphorylase* (*AGPase*) genes and interacts with *GBSS1* in the transcription and posttranslational pathways. Based on these results, the Dodamssal alleles of *SBE3* and *GBSS1* may be useful in rice breeding for fine-tuning amylose content and producing starch suited for industrial applications.

## Materials and methods

### Generation of the four near-isogenic lines

In our previous study, a recombinant inbred line (RIL) population (F_6:7_) was developed from a cross between Hwayeong (*SBE3*/*Wx^b^*) and Dodamssal (*sbe3*/*Wx^lv^*; Adeva et al., 2020). Among the RIL population, two lines (CR2121 and CR2138) harboring the Dodamssal alleles of *sbe3* and *Wx^lv^*, were crossed with Hwayeong to generate F_1_ seeds and the F_2_ plants were used for genetic analysis (Adeva et al., 2020). Among 210 F_2_ plants, four homozygous genotypes (hereafter referred to as the NIL 1, NIL 2, NIL 3, and NIL 4) were selected using two gene-specific CAPS markers (CS02_001 for *SBE3* T/C SNP and CS06_001 for *GBSS1* T/G SNP; Supplementary Figure 1; Supplementary Table 1). NIL 1 was homozygous for Hwayeong at *SBE3* (*SBE3*) and *GBSS1* (*Wx^b^*) whereas NIL 2 was homozygous for Hwayeong at *SBE3* (*SBE3*) and Dodamssal at *GBSS1* (*Wx^lv^*). NIL 3 was homozygous for Dodamssal at *SBE3* (*sbe3*) and Hwayeong at *GBSS1* (*Wx^b^*) and NIL 4 has a genotype of homozygous for Dodamssal at *SBE3* (*sbe3*) and *GBSS1* (*Wx^lv^*). For NIL 1, NIL 2, NIL 3, and NIL 4, 18, 11, four, and nine F_2_ plants were selected, respectively, to account for possible background effects. Selected F_2_ plants of NIL 1–4 were grown as lines (20 plants per line) during the F_3:4_ generation and F_5_ seeds harvested from F_4_ plants were used in this study. Plant materials used in this study were grown in the experimental field of Chungnam National University, Daejeon, South Korea in 2020 and seeds were harvested from five plants per line at 45 days after flowering and dried in the greenhouse for 2 weeks.

### DNA extraction and genotype analysis

Fresh leaves were collected from each plant and genomic DNA extraction was performed using the CTAB method as described by Causse et al. (1994) with minor modifications. PCR was conducted as described by Shim et al. (2019) with minor modifications in the amplification profile: 95°C for 5 min, followed by 35 cycles of 95°C for 30 s, 55–58°C for 30 s, and 72°C for 30 s, and 5 min at 72°C of final extension. PCR amplicons were separated on a 3% agarose gel stained with StaySafe Nucleic Acid Gel Stain (RBC, New Taipei City, Taiwan). Restriction enzymes *AccI* and *SpeI* (NEB, MA, United States) were used according to manufacturer’s instructions. Sanger sequencing of PCR products was conducted by SolGent sequencing service (SolGent Co. Ltd., Daejeon, Korea).

### Evaluation of seed morphological traits

Grain weight was measured from three plants per line with 100 randomly selected seeds per plant. Endosperm microstructure was observed using scanning electron microscopy (SEM). Transverse section of the middle of the fully matured seeds was made using a razor blade (DORCO, Seoul, Korea) and coated with platinum (Pt). Endosperms of each sample were observed using CLARA (TESCAN, Czech Republic) at 10 kV with magnification of 1,000 and 3,000x in the Center for Research Facilities (Chungnam National University, Daejeon, Korea).

### Starch isolation

Rice starch was isolated from polished rice of Dodamssal, Hwayeong, and the four NILs following the method described by Kim et al. (2020) with some modifications. Polished rice samples (10 g) were treated with 20 ml of 0.2% sodium hydroxide solution. After 2 h of shaking at room temperature at 200 rpm, samples were ground with a mortar and pestle. Thirty milliliter of 0.2% sodium hydroxide solution was added and samples were shaken for another 2 h at room temperature at 200 rpm. The resultant slurry was then filtered through 100- and 325-mesh sieves. The filtrate was centrifuged at 3,000 *g* for 20 min. The sediment was washed with distilled water (50 ml) and centrifuged at 3,000 *g* for 15 min, and these steps were repeated twice. After discarding the supernatant, the dark tailings layer atop the starch was carefully scraped and discarded. The starch was washed with water and centrifuged at 3,000 *g* for 15 min. These steps were repeated until the tailings fraction became negligible. The starch was washed with absolute ethanol and centrifuged at 3,000 *g* for 15 min and then dried at 40°C for 2 days before X-ray diffraction analysis, chain length distribution analysis, and *in vitro* starch digestibility assay.

### Chain length distribution of amylopectin in rice starch

Chain length distribution of amylopectin in rice starch was determined using a High-Performance Anion-Exchange Chromatography Coupled with Pulsed Electrochemical Detection (HPAEC-PAD) system. Sample preparation and analysis were conducted following the method described by Park et al. (2020) with minor modifications. Starch samples (10 mg) were dispersed in 2 ml 90% dimethyl sulfoxide DMSO and boiled with continuous stirring for 20 min. The solubilized starch samples were precipitated with 6 ml of absolute ethanol, and centrifuged at 2,700 rpm for 12 min. The precipitates were dissolved in 2 ml of 50 mM sodium acetate buffer (pH 3.5) and heated in a boiling water bath with continuous stirring for 20 min. After the solution was equilibrated at 37°C, isoamylase (E-ISAMY, 4.16 μl, 240 U/mg, Megazyme Co., Wicklow, Ireland) was added and the starch solution was incubated at 37°C with continuous stirring for 24 h. The enzyme was inactivated by boiling for 10 min. An aliquot (200 μl) of the debranched starch was diluted with 2 ml of 150 mM NaOH. The sample was filtered through a 0.45-μm nylon syringe filter and injected into the HPAEC-PAD on a Dionex ICS-5000 system (Dionex Co., CA, United States) consisting of a CarboPac PA200 column (3 × 250 mm). Separation was achieved using a gradient eluent with 150 mM NaOH and 0.6 M sodium acetate in 150 mM NaOH at a flow rate of 0.5 ml/min.

### X-ray diffraction

X-ray diffraction (XRD) analysis was performed using the isolated starch with X-ray diffractometer D8 ADVANCE (Bruker, Bremen, Germany) operated at 40 kV and 40 mA in the Center for Research Facilities (Chungnam National University, Daejeon, Korea). Diffractograms were obtained from 4 to 50° (2θ) at a scan rate of 2 s/step.

### Pasting characteristics

The pasting characteristics of rice flour were evaluated using rapid viscosity analysis RVA Super 4 (Newport Scientific, Sydney, Australia). Fine rice flours (3 g) of each sample were placed in an aluminum container and dispersed in 25 ml of deionized water. Samples were kept at 50°C for 1 min, and the temperature was increased from 50 to 95°C for 3.48 min and maintained at 95°C for 2.05 min. After cooling at 50°C for 3.48 min viscosity characteristics (peak, trough, breakdown, final, and setback) of the samples were evaluated. The analysis was carried out in the Department of Southern Area Crop Science, Rural Development Administration, Miryang, Korea.

### *In vitro* starch digestibility assay

*In vitro* starch digestibility assay was conducted following the methods described by Englyst et al. (1992) and Chung et al. (2009) with modifications. 450 mg of porcine pancreatic alpha-amylase (P-7545; Sigma-Aldrich Co., MO, United States) was dispersed in 4 ml of distilled water in the 50 ml of conical tube (SPL Life Science, Pocheon, Korea) and centrifuged at 1,500 *g* for 10 min. A 2.7 ml aliquot of the supernatant was transferred to a new 50-ml tube and 0.3 ml of amyloglucosidase (A-9913; Sigma-Aldrich Co., MO, United States) and 0.2 ml of invertase (E-INVPD; Megazyme Co., Wicklow, Ireland; 250 mg/ml) were added to the solution. Starch (100 mg) and 4 ml of 0.5 M sodium acetate buffer (pH 5.2) were added to each glass test tube (15 mm × 120 mm). One milliliter of enzyme solution and five glass beads (4 mm diameter) were added to each glass tube which were then incubated in a shaking water bath (37°C, 200 rpm). Aliquots (0.1 ml) were collected at each six time points (0, 15, 30, 60, 120, and 240 min) and 1 ml of 80% ethanol was added to stop the reactions. Tubes were centrifuged for 10 min with 2,000 rpm, and the glucose content was measured by supernatant of each sample. GOPOD kit (K-GLUC; Megazyme Co., Wicklow, Ireland) was used for determination of glucose content.

### Amylose and resistant starch content

All measurements and calculations in estimating the AC and RS content were performed by strictly following the Megazyme assay kit procedures (Megazyme Co., Wicklow, Ireland). For estimation of amylose content, finely crushed polished rice samples (25 mg) were completely dispersed by heating in 1 ml of dimethyl sulfoxide (DMSO). Ethanol (95%) precipitation of the starch was performed to remove lipids. The amylopectin fraction was removed by precipitation with concanavalin A followed by centrifugation. The amylose and total starch (in a separate aliquot of the acetate/salt solution) were both enzymatically hydrolyzed to D-glucose. The amylose and total starch were measured using a spectrophotometer and the absorbance was read at 510 nm against a reagent blank. For estimation of RS content, each sample (100 mg) was incubated in a shaking 37°C water bath and digested with pancreatic α-amylase and amyloglucosidase (AMG) for 16 h. The reaction was terminated by adding an equal volume of absolute ethanol and, after the centrifugation, the RS was recovered as a pellet. The RS pellets were dissolved in 2 ml of 2 M KOH with vigorous mixing in an ice-water bath with a magnetic stirrer followed by neutralization with acetate buffer. AMG was added to the solutions to hydrolyze the starch to glucose which was measured with glucose oxidase/peroxidase (GOPOD). The solution served as the measure of the RS content of each sample. The absorbance was read at 510 nm against a reagent blank.

### RNA extraction and qRT-PCR

Total RNA was extracted from panicle samples of the four NILs and two parental lines at 15 days after flowering as described by Jeon et al. (2021). Panicle samples at 15 days after flowering were used as starch-related genes are actively expressed at this stage. cDNA was synthesized from the total RNA samples and amplified using a kit (SMART GENE, Daejeon, Republic of Korea), and quantitative real-time PCR (qRT–PCR) was performed using a CFX real-time PCR system with SYBR Green Master mix (SMARTGENE, Daejeon, Korea). The rice *Ubiquitin5* (*UBQ5*) gene was used for normalization. Primers for starch-related genes developed from previous studies were used and are listed in Supplementary Table 1 (Zeng et al., 2007; Zhou et al., 2016; Baysal et al., 2020).

### Statistical analysis

One-way ANOVA, Tukey’s test, and regression analysis were conducted using Minitab19 software (https://www.minitab.co.kr/; accessed on February 3, 2022). Genetic interaction was determined by multiple regression model with two genes as independent variables and interaction term.

## Results

### Comparison of *SBE3* and *GBSS1* sequence in parental lines

In our previous study, nucleotide sequences of *SBE3* and *GBSS1* were compared using whole-genome sequencing (WGS) between Hwayeong and Dodamssal (Adeva et al., 2020). In this study, we confirmed these sequence variations using Sanger sequencing. For *SBE3* gene, one T/C SNP (T for Hwayeong and C for Dodamssal) was detected in 16th exon, which led to change of amino acid from leucine (Hwayeong) to proline (Dodamssal; Supplementary Figure 2). For *GBSS1*, Hwayeong has a *Wx^b^* allele whereas Dodamssal has a *Waxy* ancestral allele *Wx^lv^* derived from a Korean weedy rice “Gimcheonaengmi” (Adeva et al., 2020).

### *SBE3* variation changed seed morphology and starch structure in rice endosperm

**Figure 1:**
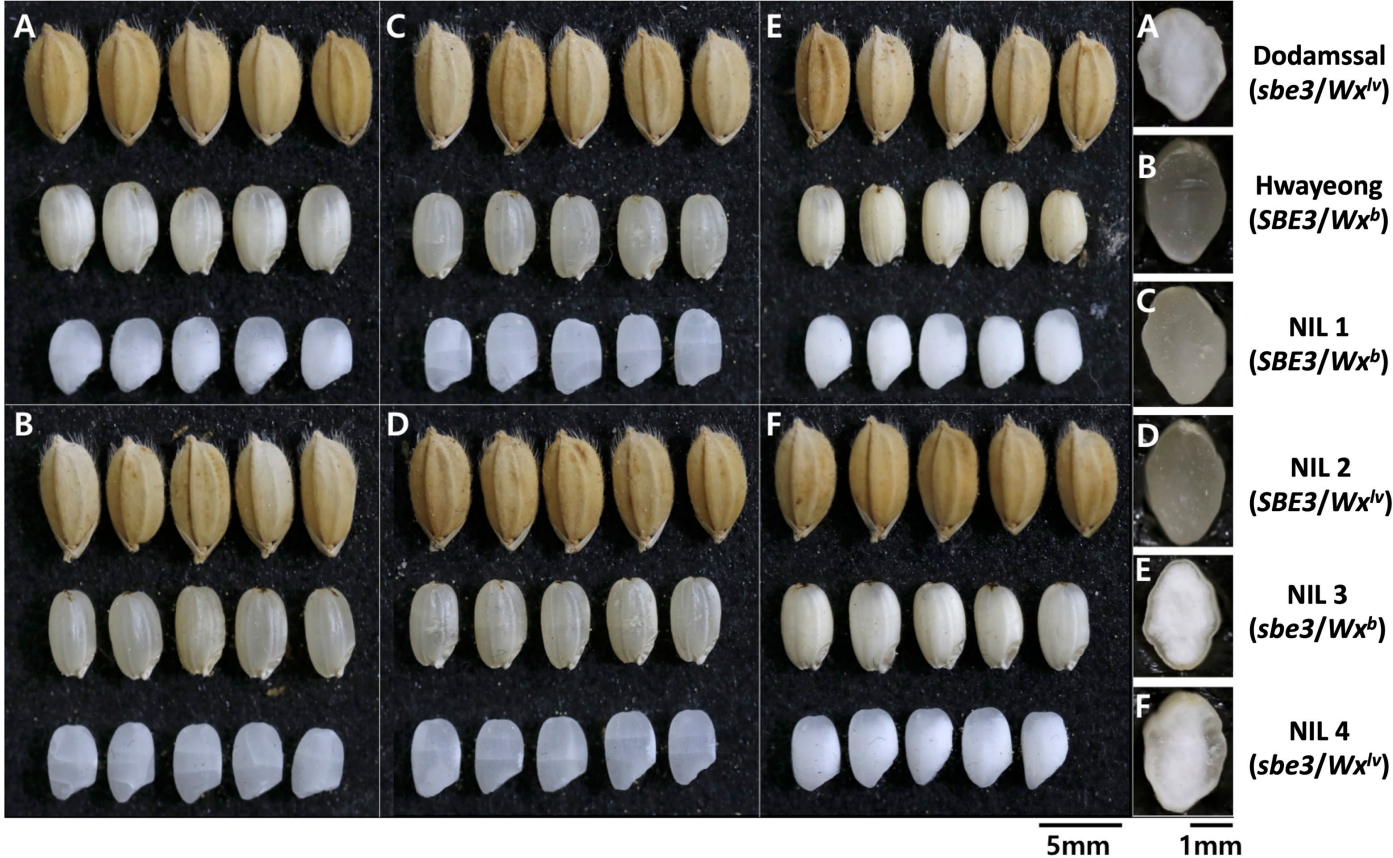
Comparison of seed morphology of two parental lines and four near-isogenic lines (NILs). **(A)** Dodamssal, **(B)** Hwayeong, **(C)** NIL 1 (*SBE3*/*Wx^b^*), **(D)** NIL 2 (*SBE3*/*Wx^lv^*), **(E)** NIL 3 (*sbe3*/*Wx^b^*), and **(F)** NIL 4 (*sbe3*/*Wx^lv^*).

Seeds were compared between the four NILs and parental lines (Hwayeong and Dodamssal; Figure 1). Grains of Dodamssal, NIL 3, and NIL 4, all of which harbor *sbe3* alleles, exhibited an opaque and floury endosperm, while a translucent endosperm was observed in grains of Hwayeong, NIL 1, and NIL 2, which carry *SBE3* alleles. This observation suggests that variation in *SBE3* was associated with change in endosperm morphology.

**Figure 2:**
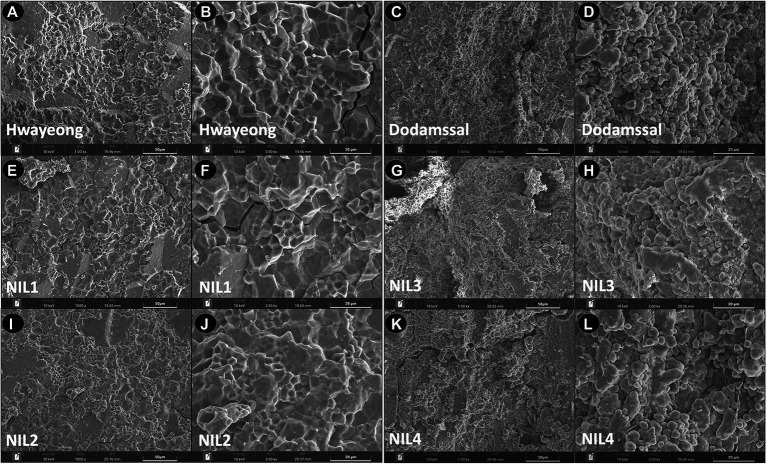
Ultrastructure of endosperm surface structure observed by scanning electron microscope in two parental lines and four NILs. Hwayeong **(A,B)**, Dodamssal **(C,D)**, NIL 1 **(E,F)**, NIL 2 **(I,J)**, NIL 3 **(G,H)**, and NIL 4 **(K,L)** with two different magnifications (x 1,000 and x 3,000).

To observe the starch granule structure of the grains, SEM analysis was conducted (Figure 2). Hwayeong, NIL 1, and NIL 2 showed polygonal starch granules with sharp edges, smooth flat surfaces, and compound starch granules (Figure 2). However, the endosperms of Dodamssal, NIL 3, and NIL 4 were filled with rounded, irregularly shaped starch granules with air spaces between granules (Figure 2). No other obvious differences were observed between NIL 1 and NIL 2, and NIL 3 and NIL 4, indicating that the variation in *SBE3* might be a major determinant of the difference in seed morphology and granule structure between Hwayeong and Dodamssal.

One hundred grain weight (100-GW) was also measured, and NIL 1, NIL 2, NIL 3, and NIL 4 showed 2.48, 2.62, 2.15, and 2.49 g 100-GW, respectively (Supplementary Figure 3A). NIL 2 had a significantly higher 100-GW among genotypes while NIL 3 had a significantly lower 100-GW than the other genotypes. Although NIL 4 has an *sbe3* allele, 100-GW of NIL 4 was significantly higher than NIL 3. This could be explained by the presence of the *Wx^lv^* in NIL 4. The four NILs did not show significant differences in other agronomic traits, such as grain fertility, plant height, panicle number, and panicle length (Supplementary Figures 3B–E). These results suggest that the progenies from two *japonica* lines are similar in agronomic traits except 100-GW and starch-related traits. It is also possible that the background effects were neutralized by selecting 4–18 F_2_ plants per each NIL for evaluation.

### *SBE3* Dodamssal allele led to increase in long amylopectin chains

**Figure 3:**
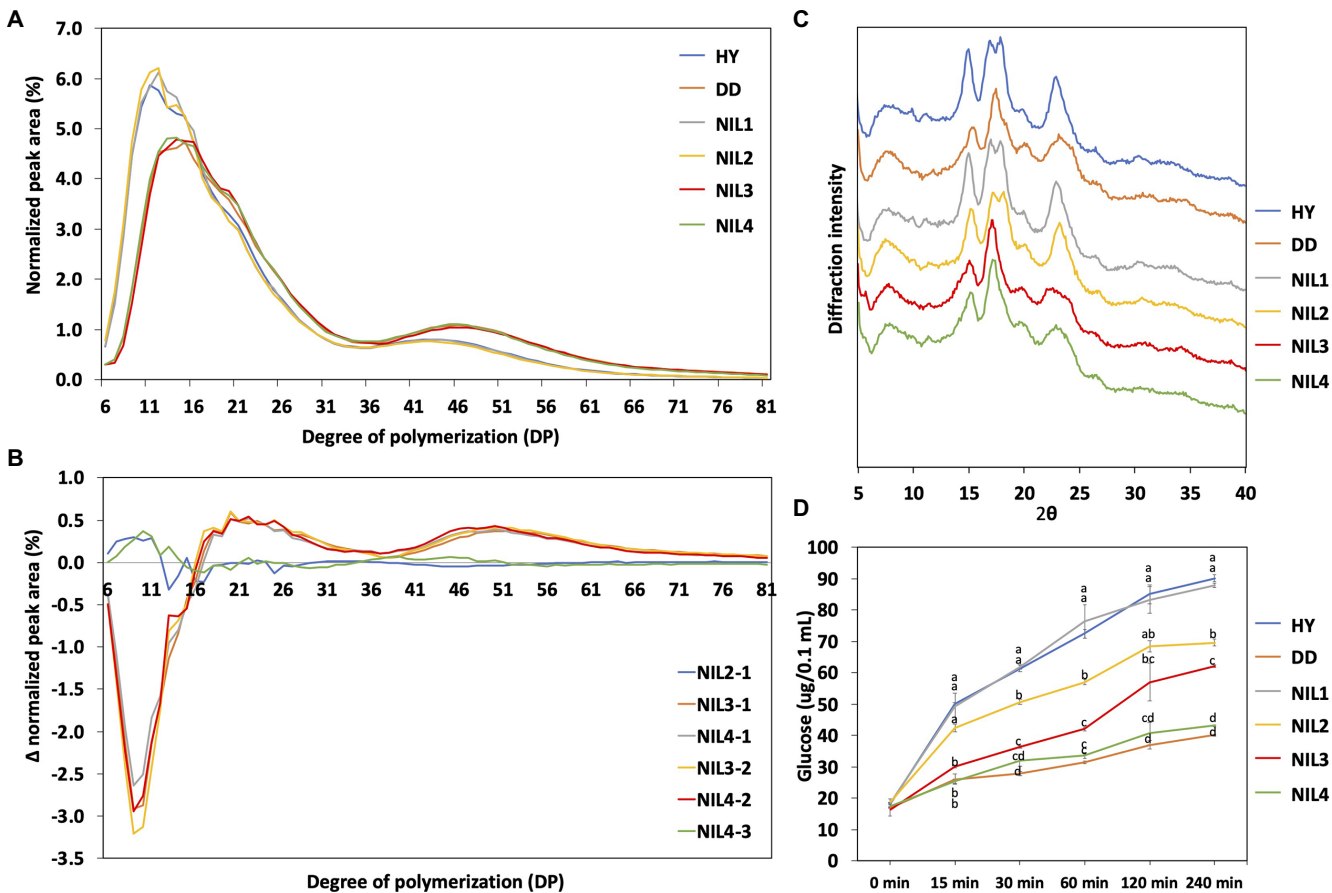
Physico-chemical characteristics of four NILs. **(A)** Chain length distribution of the two parental lines and four NILs. **(B)** Differences in the molecular structure of amylopectin between the four NILs. Differences are shown as Δ (Delta) normalized peak area. **(C)** X-ray diffraction pattern of rice starch samples. **(D)** Starch digestibility analysis of the two parental lines and four NILs. Glucose content was determined by GOPOD kit (Megazyme Co., Wicklow, Ireland) from the digestion of each starch sample. The same letter is not significantly different at *p* = 0.05 based on Tukey’s test. HY, Hwayeong; DD, Dodamssal.

Chain length distribution of amylopectin was examined to understand the difference in amylopectin structure (Figure 3A). The six genotypes were classified into two groups based on their patterns of chain length distribution. Hwayeong, NIL 1, and NIL 2 composed one group, and the other group included Dodamssal, NIL 3, and NIL 4. This result indicates that the sequence variation in *SBE3* greatly affected amylopectin chain length distribution, but variation in *GBSS1* did not. Despite Dodamssal and NIL 4 having higher AAC than NIL 3, the amylopectin chain length distribution patterns of the three genotypes were similar. To observe the detailed differences in chain length distribution, each value of four NILs was subtracted showing two major different distribution patterns and Δ (Delta) normalized peak area (Figure 3B). For example, NIL 2-1 is the value obtained by subtracting NIL 1 from NIL 2. NIL 3-1, NIL 4-1, NIL 3-2, and NIL 4-2 showed similar distribution pattern while NIL 2-1 and NIL 4-3 had different distribution patterns overlapping to each other. Comparison of NIL 3-1, NIL 4-1, NIL 3-2, and NIL 4-2 indicated the effect of *SBE3* variation and the subtraction curve of this group showed a decrease in the proportion of shorter chains, degree of polymerization (DP) 6-16 with an increase in the fraction of longer chains (DP 17–81). Both NIL 2-1 and NIL 4-3 displayed an increase in DP 6–12, but the difference was insignificant. Amylopectin chains were classified into four groups (A, B_1_, B_2_, and B_3_) based on the amylopectin cluster model (Hanashiro et al., 1996). Dodamssal, NIL 3, and NIL 4 exhibited significantly higher average chain length and distribution of B_2_ and B_3_ chains, while Hwayeong, NIL 1, and NIL 2 showed significantly increased A chains (Supplementary Table 2). These results indicate that the Dodamssal *sbe3* allele conferred a change in the amylopectin structure.

### Starch crystallinity of four NILs

To examine the crystal structure of the rice starch in parental lines and the four NILs, XRD analysis was conducted (Figure 3C). Starch crystallinity is classified into three types: A-type, B-type, and C-type, a hybrid of A-type and B-type based on its XRD pattern (Park et al., 2020). Hwayeong, NIL 1, and NIL 2 showed continuous double diffraction peaks at 17 and 18° 2θ, and strong diffraction peak at 15 and 23° 2θ. Based on these results, these three genotypes were classified as having an A-type starch. However, a strong single diffraction peak was detected at near 17° 2θ in Dodamssal, NIL 3, and NIL 4, and relatively weak peaks were found at 15 and 23° 2θ, indicating that these lines could be classified as having B-type starch crystals. These results indicated that *SBE3* plays an important role in rice starch crystal structure.

### Different starch viscosity properties in four NILs

To investigate the viscosity properties of rice starch, a Rapid Visco-Analyser (RVA) was employed (Table 1). Significant differences were observed in all properties. NIL 3 showed the highest pasting temperature, final viscosity, and peak time. Dodamssal and NIL 4 had similar starch viscosity properties, and pasting temperature of Dodamssal and NIL 4 were higher than those of Hwayeong, NIL 1, and NIL 2. Hwayeong and NIL 1 displayed the highest peak viscosity. Peak viscosity of NIL 3 with the *sbe3* Dodamssal allele was lower than the other three NILs. Although NIL 2 and NIL 3 had similar amylose content, their starch viscosity traits were considerably different suggesting that the variation in *SBE3* and *GBSS1* alleles affects the starch viscosity properties.

**Table 1:** Pasting properties of rice flours analyzed by rapid visco-analyzer.

	Pasting temperature (°C)	Peak viscosity (RVU)	Trough viscosity (RVU)	Breakdown^1)^ (RVU)	Final viscosity (RVU)	Setback^2)^ (RVU)	Peak time (min)
Dodamssal	84.3 ± 0.1 b	233.0 ± 3.5 d	178.3 ± 7.1 ab	54.7 ± 3.6 c	290.4 ± 4.2 cd	57.5 ± 7.7 bc	5.6 ± 0.1 b
Hwayeong	75.5 ± 0.0 c	449.9 ± 2.0 a	185.6 ± 4.7 a	264.4 ± 4.0 a	299.8 ± 5.7 bc	−150.1 ± 4.7 a	5.6 ± 0.1 b
NIL 1	74.7 ± 0.1 c	440.6 ± 0.4 b	178.6 ± 2.5 ab	262.0 ± 2.9 a	301.4 ± 3.4 bc	−139.3 ± 3.9 a	5.6 ± 0.0 b
NIL 2	71.7 ± 0.5 d	262.4 ± 4.7 c	144.9 ± 1.2 c	117.5 ± 3.6 b	307.5 ± 2.4 b	45.1 ± 2.6 b	5.6 ± 0.0 b
NIL 3	87.6 ± 0.0 a	196.0 ± 4.0 f	185.0 ± 3.7 a	11.0 ± 0.4 d	341.0 ± 5.3 a	145.0 ± 1.4 d	6.3 ± 0.0 a
NIL 4	83.7 ± 0.7 b	221.0 ± 0.6 e	169.9 ± 0.8 b	51.0 ± 1.3 c	286.3 ± 5.9 d	65.4 ± 5.5 c	5.4 ± 0.2 b

Data are represented as mean values ± standard deviation. Mean values that do not share the same letters are significantly different at *p* = 0.05 based on Tukey’s test.

1) Peak viscosity minus trough viscosity.

2) Final viscosity minus peak viscosity.

### High amylose and resistant starch in Dodamssal is associated with low starch digestibility

To investigate the effect of variation in *SBE3* and *GBSS1* on starch digestibility, digestibility assays were performed as shown in Figure 3D. The glucose content of the digested starch samples was measured at six time intervals each (0, 15, 30, 60, 120, and 240 min). Hwayeong and NIL 1 had the highest increase in glucose content. NIL 2 was lower than that of Hwayeong and NIL 1, but higher than that of NIL 3. Although NIL 2 and NIL 3 had similar AAC, the starch of NIL 2 was more easily digested than NIL 3. This result might be due to different RS contents between NIL 2 and NIL 3 (see below). NIL 4 and Dodamssal showed the lowest starch digestibility due to high amylose content and RS content. These data suggest that the combination of two alleles *sbe3* and *Wx^lv^* would be valuable for non-digestible starch rice breeding program.

### Genetic interaction of *SBE3* and *GBSS1* in amylose and resistant starch content

**Figure 4:**
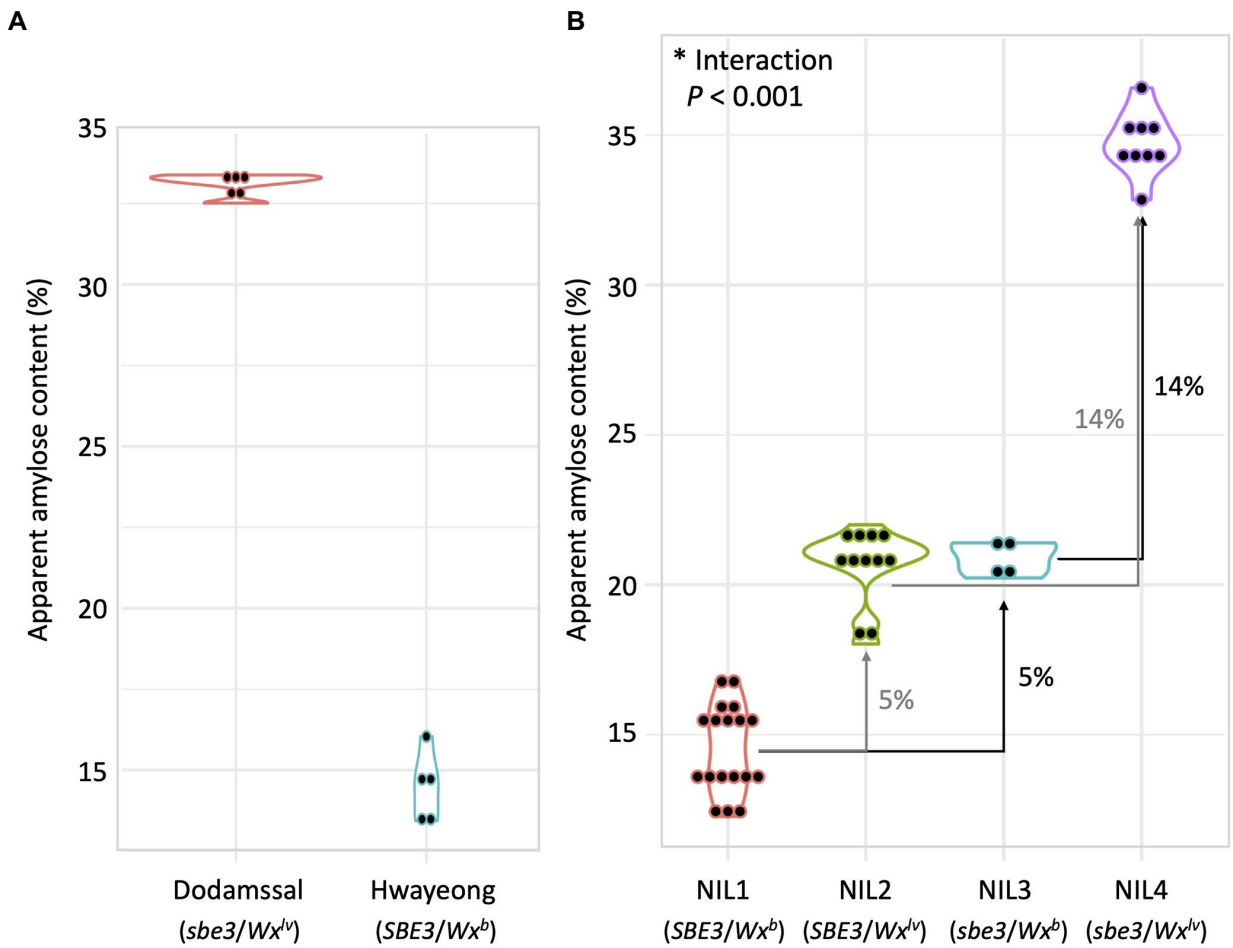
Comparison of apparent amylose content (AAC) between **(A)** Dodamssal and Hwayeong, **(B)** four NILs. Violin plots were used to show distribution of data points. The numbers beside the arrows indicate increased AAC.

Apparent amylose content of Hwayeong, Dodamssal, and the four NILs were compared (Figure 4). Dodamssal and Hwayeong showed about 33 and 15% AAC, respectively (Figure 4A). AAC values about 15, 21, 21, and 35% were observed in NIL 1, NIL 2, NIL 3, and NIL 4, respectively, indicating that the Dodamssal alleles of *SBE3* and *GBSS1* (*sbe3* and *Wx^lv^*) significantly enhanced the AAC (Figure 4B). Replacement of *Wx^b^* in NIL 1 with *Wx^lv^* NIL 2 at *GBSS1* corresponded to a 5% increase of AAC, while the difference in AAC between NIL 3 and NIL 4 was about 14%. To determine the genetic interaction between *SBE3* and *GBSS1*, a multiple regression model was employed. Significant interaction was found between two genes (*p* < 0.001, Supplementary Table 3), and the Dodamssal alleles of *SBE3* and *GBSS1* synergistically increased the AAC in the Hwayeong genetic background, which was consistent with our previous observation (Adeva et al., 2020).

Resistant starch content was also measured from rice flours of Hwayeong, Dodamssal, and the four NILs. Hwayeong, NIL 1, and NIL 2 had 0.07, 0.07, and 0.30% RS content, respectively (Supplementary Figure 4). While the RS content of NIL 2 was higher than those of Hwayeong and NIL 1, the difference was not significant. Dodamssal and NIL 4 exhibited similar RS content of 8.17 and 7.55%, respectively. NIL 3 had 11.51% RS content and was significantly higher than Dodamssal and NIL 4 (*p* < 0.001). Despite NIL 3 and NIL 4 both carrying the Dodamssal *sbe3* allele, the RS content of NIL 3 was higher than that of NIL 4 suggesting an interaction between *SBE3* and *GBSS1*. Multiple regression analysis showed a significant interaction between *SBE3* and *GBSS1* in regulating RS (*p* < 0.001, Supplementary Table 4). Further study is needed to understand the association between *SBE3* and *GBSS1* in the regulation of the RS content.

### Dynamic changes in starch-related genes expression

**Figure 5:**
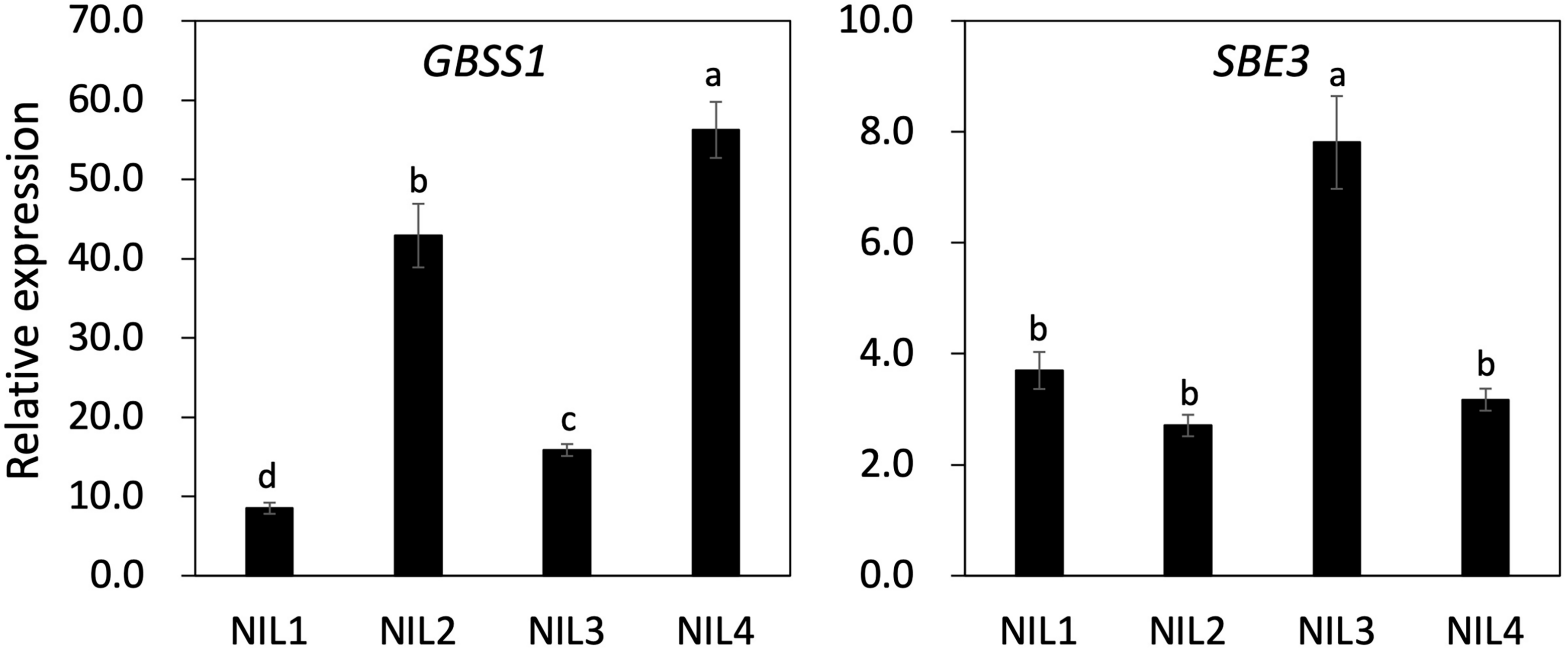
Gene expression of *GBSS1* and *SBE3* in four NILs using panicles samples from 15 days after flowering. *UBQ5* was used for gene normalization. The same letter on the bar is not significantly different at *p* < 0.05 based on Tukey’s test, respectively.

To understand the genetic interaction of two genes (*GBSS1* and *SBE3*) on AAC and the physico-chemical properties of the four NILs, gene expression profiling was carried out using panicle samples at 15 days after flowering (Figure 5; Supplementary Figure 5). Significant differences were observed in expression level of *GBSS1* between the NILs. NIL 4 showed the highest gene expression consistent with AAC and the *GBSS1* expression level in NIL 4 was 6.7, 1.3, and 3.5 times higher than that observed for NIL 1, NIL 2, and NIL 3, respectively. Although NIL 2 and NIL 3 displayed similar AAC, expression of *GBSS1* in NIL 2 was 2.7 times higher than NIL 3. *SBE3* expression in NIL 3 was significantly higher (about 2-fold) than the other genotypes. Paralogous isoforms of *GBSS1* and *SBE3* expression were also examined (Supplementary Figure 5A). The expression level of *GBSS2* and *SBE1* was upregulated in NIL 3 and NIL 4 compared to NIL 1 and NIL 2. NIL 4 showed the highest *SBE4* expression level, which was significantly different from the other NILs.

The expression of other starch-related genes including *ADP-glucose pyrophosphorylase* (*AGP*; Supplementary Figure 5B), *soluble starch synthase* (*SS*; Supplementary Figure 5C), *starch debranching enzyme* [*Pullulanase* (*PUL*) and *Isoamylase* (*ISA*); Supplementary Figure 5D], and *starch phosphorylase* (*PHOL* and *PHOH*; Supplementary Figure 5E) was examined. NIL 3 and NIL 4 or NIL 3 alone showed significantly higher expression of the *soluble starch synthase* isoforms compared to NIL 1 and NIL 2. Moreover, NIL 3 had higher expression of *SSI*, *SSIIa*, *SSIIc*, *SSIIIa*, *SSIIIb*, and *SSIVb* than NIL 4. Similar gene expression patterns were also observed for the *AGPase* genes and *starch debranching enzyme* genes. The transcript levels of *AGPS1*, *AGPS2a*, *AGPS2b*, and *AGPL2* were higher in NIL 3 than NIL 1 and NIL 2.

The transcript level of *GBSS1* and *SBE3* was examined in Hwayeong and Dodamssal (Supplementary Figure 6). Dodamssal showed significantly higher expression level of *GBSS1*, while *SBE3* expression was not significantly different between two parental lines. Dodamssal also displayed significantly higher expression in two major starch biosynthesis genes, *SSI* and *SS3a,* than Hwayeong. For *SBE* paralogous genes, no significant difference between two lines was not observed in *SBE1* whereas Hwayeong showed higher *SBE4* level than Dodamssal.

Transcript profiling indicates that the different alleles and allele combinations of *GBSS1* and *SBE3* changed the expression level of not only *GBSS1* and *SBE3* but also other starch-related genes that might be responsible for the increased AAC and altered physico-chemical characteristics of the rice starch in the NILs.

## Discussion

The functional rice variety “Dodamssal” with high amylose and resistant starch content (RS) is widely used for products such as rice noodles (Cho et al., 2019; Park et al., 2020). However, Dodamssal has weaknesses such as lacks of disease and insect pest resistances and low-yield. The program to develop new versions of Dodamssal with high-yield potential and resistance to diseases is underway using the marker assisted selection (Cho et al., 2019). In our previous study, we determined that two major starch biosynthesis genes, *SBE3* and *GBSS1* are responsible for the high amylose and RS in Dodamssal (Adeva et al., 2020). However, the molecular mechanism how these two genes interact at the molecular level and alter the physico-chemical properties of rice starch remained to be determined. In this study, we developed four near-isogenic lines carrying different allelic combinations of two major starch biosynthesis genes, *SBE3* and *GBSS1,* to understand the genetic interaction and how these two genes alter the physico-chemical properties of rice starch.

Analysis of the seed morphology and starch granule structure of the NILs and parental lines confirmed the association of these traits with the variation in *SBE3* (Figures 1, 2). These results are consistent with previously reported *SBE3* mutants, EM10 and Jiangtangdao 1, which also showed a similar floury endosperm phenotype (Yang et al., 2012). In addition to differences in their endosperms, grain weight (100-GW) variation was observed among the four NILs with NIL 3 showing significantly lower 100-GW than the other three NILs (Supplementary Figure 3A). Nishi et al. (2001) found that *SBE3* (*Ae*) gene dosage was associated with grain weight and size. The *ae* allele dosage in the endosperm decreased grain weight and grain size with a decrease in the relative BE2b protein content. Yang et al. (2016) also showed that *SBE3* overexpression of transgenic T_1_ plants generated in the Jiangtangdao 1 (*sbe3*) genetic background had significantly higher 1,000-seed weight than control plants. The significantly higher 100-GW of NIL 2 than NIL 1 appears to be due to the presence of *Wx^lv^* in NIL 2. This result is consistent with that of the previous study that the *Wx^lv^* allele explained 14.6% (*p* < 0.001) of the phenotypic variation of the grain weight in the RIL population (Adeva et al., 2020). Although these results suggest the association of *Wx^lv^* with grain weight, it remains to be determined whether *Wx^lv^* or a tightly linked QTL contributed to an increase in grain weight.

Variations in *SBE3* and *GBSS1* significantly altered the physico-chemical properties of rice starch. Of these two genes, variation in *SBE3* strongly affected the starch properties. For X-ray diffraction analysis, Hwayeong, NIL 1, and NIL 2, harboring an *SBE3* allele, showed an A-type diffraction pattern, while Dodamssal, NIL 3, and NIL 4, harboring an *sbe3* allele, displayed a B-type XRD pattern. Park et al. (2020) reported that Dodamssal displayed a C-type X-ray diffraction pattern with a predominant B-type (CB-type) based on the broad peak at 22–23° 2θ without shoulder peak at 18° 2θ. Similar diffraction patterns were observed in Dodamssal, NIL 3, and NIL 4. However, the diffraction peak of NIL 3 at 22–23° 2θ was slightly broader than Dodamssal and NIL 4. The *SBE3* (*BEIIb*) mutant EM10 and the *be1/be2b* double mutant were classified as having B-type crystal structure (Miura et al., 2021). Jiangtangdao1, which harbors the same T/C mutation in *SBE3* as NIL 3, also had a B-type diffraction pattern (Yang et al., 2016). The donor of the *sbe3* allele, “Goami2” was reported as a B-type starch (Yoon et al., 2013). These studies consistently confirmed that mutation in *SBE3* led to a B-type or a CB-type crystal structure.

With regard to starch viscosity properties, NIL 3 had the highest pasting temperature, final viscosity, and peak time values although NIL 4 harbors the same *sbe3* allele and has higher AAC than NIL 3. NIL 3 requires more energy and time for gelatinization, which may be associated with a higher RS and the amylose-lipid complexes found in resistant starch. When the thermal properties of starch in the *be1*, *be2b*, and *be1be2b* mutants were analyzed, the onset, peak, and conclusion gelatinization temperature were associated with resistant starch and amylose contents (Miura et al., 2021). It was previously demonstrated that *Wx^lv^* affected eating and cooking quality of rice grains by modulating the size of amylose molecules (Zhang et al., 2019). Thus, presence of *Wx^lv^* in NIL 4 may explain the difference in starch viscosity traits compared to NIL 3.

Variations in *GBSS1* and *SBE3* also changed the amylopectin chain length distribution (Figure 3B). The comparison of amylopectin chain length distribution between NIL 2–1 and NIL 4–3 revealed that *Wx^lv^* was associated with an increase in amylopectin short chains (DP 6–12), consistent with a previous observation (Crofts et al., 2012). Moreover, a T/C SNP in *SBE3* was associated with a decrease in short chains (DP 6–16) and an increase in long chains (DP 16–81). In particular, the distribution patterns of NIL 3-1, NIL 4-1, NIL 3-2, and NIL 4-2 were consistent with those of previous reports on the distribution pattern between EM10 and wild type (Nishi et al., 2001; Miura et al., 2021).

Deficiencies in starch synthases or starch branching enzymes can significantly increase the AAC. Two *SS3a* mutant lines, a *Tos17* insertion and T65 MNU mutant, displayed an increased *GBSS1* protein expression level in the developing seeds, and *GBSS1* mRNA was upregulated in *Tos17* insertion mutant compared to the wild type (Fujita et al., 2007). The *GBSS1* protein level of *ss3a*, *be2b*, and *ss3a/be2b* deficiency mutants slightly increased compared to their wild type counterparts (Asai et al., 2014). In addition, a significant interaction was reported between *ss3a* and *Wx* allele in starch synthesis, and the *ss3a* mutant in the *indica* (*Wx^a^*) genetic background showed higher amylose content or resistant starch than the *ss3a* mutant in *japonica* (*Wx^b^*) rice (Crofts et al., 2012; Zhou et al., 2016). The relative GBSS1 content was enhanced in *ss3a*/*Wx^b^* than *SS3a*/*Wx^b^* genotype, indicating a loss-of-function of *SS3a* led to upregulation of the *GBSS1* protein level (Crofts et al., 2012). Synergistic increase of the AAC was observed when the different genotype combinations of *SS3a* and *GBSS1* were compared (Crofts et al., 2012). The *ss3a*/*wx^a^* line showed the highest AAC (41.3%), and two alleles *ss3a* and *Wx^a^* contributed to an increase in the AAC compared to their counterparts *SS3a* and *Wx^b^*, respectively. Interestingly, the difference in the GBSS1 protein expression between *SS3a*/*Wx^a^* and *ss3a*/*wx^a^* genotype was not significant and the synergistic effect was possibly due to the elevated AGPase activity (Crofts et al., 2012). The expression level of *GBSS1* mRNA or protein in *ss3a* mutant developed by Zhou et al. (2016) was not significantly different with wild type, indicating that interaction possibly occurred at the posttranslational level (Zhou et al., 2016). It is apparent that the genetic interaction might exist between starch synthase genes and *GBSS1* in regulating amylose content.

In the present study, the *sbe3* allele led to significantly increased amylose content and *GBSS1* gene expression (Figures 4, 5). NIL 3 (*sbe3*/*Wx^b^*) showed 21% AAC and 1.9 times higher *GBSS1* transcript level than NIL 1 (*SBE3*/*Wx^b^*), and NIL 4 (*sbe3*/*Wx^lv^*) has 35% AAC with 6.6 times higher *GBSS1* expression than NIL 1 (*SBE3*/*Wx^b^*). The data combined from this study and previous reports suggest that *SBE3* directly or indirectly regulates the expression of starch synthase and *AGPase* genes including *SS3a* and interacted with *GBSS1* in the transcriptional and posttranslational pathways. However, more specific mechanisms need to be examined to fully validate the possible genetic interactions among *SBE3*, *SS3a*, and *GBSS1*. Another genetic interaction between *SBE3* and *GBSS1* was observed in the regulation of RS content (Supplementary Figure 4; Supplementary Table 4). *Wx^lv^* decreased the RS content in the *sbe3* genetic background [comparison between NIL 3 (*sbe3*/*Wx^b^*) and NIL 4 (*sbe3*/*Wx^lv^*)] while *Wx^lv^* did not significantly change the RS content in the *SBE3* genetic background [comparison between NIL 1 (*SBE3*/*Wx^b^*) and NIL 2 (*SBE3*/*Wx^lv^*)]. A similar result was observed from a previous study. Goami2, one of the parental lines of Dodamssal, has the *sbe3* and *Wx^b^* alleles which is of same genotype with NIL 3 (Adeva et al., unpublished data) and displayed 17.71% RS content (Cho et al., 2019). However, the RS content of Dodamssal was 13.62%, indicating the existence of genetic interaction between two genes in controlling RS content in rice (Cho et al., 2019). The interaction could be caused by substrate competition between amylose and RS biosynthesis. Further study is required to understand the genetic mechanisms in regulating starch content and their interactions.

Previously, studies have focused on identifying single genes controlling traits of agronomic importance. To better understand how the quantitative traits are regulated, QTL-QTL or gene–gene interactions need to be studied using genetically homogeneous populations, such as chromosome substitution, introgression, and near-isogenic lines to reduce background noises (Mackay, 2014). In the present study, we developed near-isogenic lines derived from a cross between two closely related *japonica* varieties. Because of their genetic closeness between two parental lines, the interaction between two genes, *SBE3* and *GBSS1*, was clearly examined without interference of other genetic factors. NILs for various starch-related genes with different alleles will be necessary to better define the genetic relationships between starch-related genes.

## Data availability statement

The original contributions presented in the study are included in the article/Supplementary material, further inquiries can be directed to the corresponding author.

## Author contributions

K-CS, CA, and S-NA designed the experiments and wrote the manuscript. TT edited the manuscript and provided advice on the experiments. J-WK and J-HC performed physico-chemical trait evaluation. H-SL, HK, and NL conducted the agronomic traits investigation and qRT-PCR analysis. All authors contributed to the article and approved the submitted version.

## Funding

This work was carried out with the support of “Cooperative Research Program for Agriculture Science and Technology Development (Project No. PJ015757)” Rural Development Administration, Republic of Korea.

## Conflict of interest

HK was employed by the company LG Chem., Ltd.

The remaining authors declare that the research was conducted in the absence of any commercial or financial relationships that could be construed as a potential conflict of interest.

## Publisher’s note

All claims expressed in this article are solely those of the authors and do not necessarily represent those of their affiliated organizations, or those of the publisher, the editors and the reviewers. Any product that may be evaluated in this article, or claim that may be made by its manufacturer, is not guaranteed or endorsed by the publisher.

## Supplementary material

The Supplementary material for this article can be found online at: https://www.frontiersin.org/articles/10.3389/fpls. 2022.968795/full#supplementary-material

## Abbreviations

SBE: Starch branching enzyme
GBSS1: Granule binding starch synthase 1
SS: Soluble starch synthase
DBE: Starch debranching enzyme
ISA: Isoamylase
PUL: Pullulanase
AGP: ADP-glucose pyrophosphorylase
NILs: Near-isogenic lines
QTL: Quantitative trait loci
SNP: Single nucleotide polymorphism
XRD: X-ray diffraction
AAC: Apparent amylose content
RS: Resistant starch
