# Supplementary material for: Interaction of starch branching enzyme 3 and granule-bound starch synthase 1 alleles increases amylose content and alters physico-chemical properties in japonica rice (Oryza sativa L.)
Source: Front Plant Sci. 2022 Aug 5;13:968795. doi: 10.3389/fpls.2022.968795 (PMC9389286; doi:10.3389/fpls.2022.968795)
Supplement: Supplementary file 2 [file Data_Sheet_2.docx]

**Supplementary Fig. 1.** Graphical genotypes of four near-isogenic lines (NILs). White regions indicate Hwayeong genetic background and black regions indicate Dodamssal chromosomal segments.

**Supplementary Fig. 2**. *SBE3* and *GBSS1* gene structures and the location of SNPs between Hwayeong and Dodamssal.

**Supplementary Fig. 3**. Comparison of agronomic traits of the four NILs. (**A**) Grain weight, (**B**) Seed fertility, (**C**) Plant height, (**D**) Panicle numbers, and (**E**) Panicle length.

**Supplementary Fig. 4**. Investigation of resistant starch content using rice flours from the parental lines and four NILs. (**A**) Comparison of resistant starch content. ^*^The letters indicated significant difference based on the Tukey’s test at *P* < 0.05.  (**B**) Color intensity of resistant starch content measured by Megazyme kit (Megazyme Co., Wicklow, Ireland). HY: Hwayeong, DD: Dodamssal.

**Supplementary Fig. 5**. Gene expression profiling of starch-related genes with qRT-PCR using panicle samples at 15 days after flowering. *UBQ5* was used for gene normalization. The letters indicated significant difference based on the Tukey’s test at *P* < 0.05. (**A**) Paralogous genes of *GBSS1* and *SBE3*, (**B**) *ADP-glucose pyrophosphorylase* (*AGP*), (**C**) *soluble starch synthase* (*SS*), (**D**) *starch debranching enzyme* (*Pullulanase* (*PUL*) and *Isoamylase* (*ISA*)), and (**E**) *starch phosphorylase* (*PHOL* and *PHOH*).

**Supplementary Fig. 5**. (Continued)

**Supplementary Fig. 6**. Comparison of the expression level of major starch biosynthesis genes between Hwayeong (HY) and Dodamssal (DD). 15 days after flowering panicle samples were used and UBQ5 was used for gene normalization. Asterisk (**) indicated significant difference at *P* < 0.01 based on the student’s *t*-test.
